# Revealed consumers’ preferences for fresh produce attributes in Chinese online markets: A case of domestic and imported apples

**DOI:** 10.1371/journal.pone.0270257

**Published:** 2022-06-24

**Authors:** H. Holly Wang, Xiao Han, Yu Jiang, Guoyong Wu

**Affiliations:** 1 Department of Agricultural Economics, Purdue University, West Lafayette, IN, United States of America; 2 New Hope Group, Chengdu, Sichuan, China; 3 The West Center for Economic Research, Southwestern University of Finance and Economics, Chengdu, Sichuan, China; 4 Guizhou Grassroots Social Governance Innovation High-end Think Tank, Ecological Civilization (Guizhou) Research Institute, Guiyang, Guizhou, China; 5 School of Economics, Guizhou University, Guiyang, Guizhou, China; 6 Rural Revitalization Research Institute in Karst Region of China, Guizhou University, Guiyang, Guizhou, China; University of Almeria, SPAIN

## Abstract

**Background:**

The online market is getting popular today, and consumers’ preferences about products are revealed differently in online and offline markets. Especially, fresh food purchasing online is very different from non-food products due to its unique features such as perishability, low cost and frequent purchases, low value-volume ratio, and highly relevance to safety and health. However, studies on online fresh food are rather few, and this study will fill the gap by investigating consumers’ preferences for fresh food online purchasing.

**Methods:**

Using unique data observed from online stores, we conduct a hedonic analysis of fresh produce online market using apple market in China, avoiding hypothetical bias. Propensity Score Matching is used to check the online promotion effects. The data are apples transactions from Jingdong JD e-commerce platform with sample size 8,200, observed across six weeks from 11/26/2018 to 12/31/2018. Variables used include prices, promotions, varieties, places of origin, fruit size, labeled as organic or green food, watercore label, and customer reviews for the products as well as for the venders.

**Results:**

We found place of origin, food safety and eco-certificates, and sensory features all influence apple prices which reflect consumer preferences. In addition to product features, store features such as former customers’ review for the store, a video post of the product, and other latent product features through former customers’ review for the product quality also influence consumers’ preference reflected by price.

**Conclusions:**

In additional to product intrinsic features, consumer show preferences and valuation for online market special features.

## Introduction

Shopping online has become increasingly popular in recent years in the world. There are some fundamental differences between shopping fresh food products online or offline. While originally stated with non-food products such as books and CDs, E-commerce has also expanded to various food products, among which fresh foods are relatively new and challenged because of their nature of perishability and low value-to-volume ratio. Unlike buying non-food products that verbal and visual description can usually give sufficient information about the product quality, the touch-and-feel experience for consumers to select food product, which is common in offline stores, is not available in online stores [1]. In spite of the lack of the touch-and-feel experience, online consumers can get access to more comprehensive information about products, both price and non-price attributes [2]. In addition, online consumers can also obtain explicit information about the online stores’ characteristics, such as the ratings and reviews from former consumers, in addition to its convenience over shopping at offline stores [1,3].

China is the biggest online market among all countries. According to China’s National Bureau of Statistics [4], online retail sales in China reached a record high of 7.18 trillion yuan in 2017, or $1.07T, an annual increase of 32.2% from the previous year. This dramatic growth is benefited from China’s fast adoption of smartphones, high residence density, efficient logistics for delivery with low cost and Chinese e-commerce giants’ efforts to promote e-commerce [5–7]. Currently, the Chinese e-commerce market is dominated by Alibaba and JD.com Inc., with market shares of 58.2% and 16.3%, respectively [8]. Studies on Chinese fresh food online markets are rather limited [9]. With tremendous differences between shopping food products online and offline, consumers’ preferences perceived by sellers and reflected on the pricing of the products in the two forms of shopping is different. Among the limited studies on online markets, they mostly focus on the product-related attributes: one study on olive oil sold online found significant effects of extrinsic attributes on the price [10]; and another recent study using hedonic pricing model found a price premium of green food certificate in online stores but not in offline stores suggesting a difference in pricing mechanism between online and offline markets [11]; also a study reveals there is a significant and positive impact of place-of-origin on price premium for agricultural products sold online [12]. However, these studies did not include store-related attributes, which can also play a role in the pricing of the products [13–15]. Another gap in the literature for consumer preference of online fresh food shopping is that most existing studies are based on stated preference using survey data [16,17], or using home scan data [9] without any store attributes. Further, there exist many studies on consumers’ willingness-to-pay (WTP) for food attribute labels that are of healthy food, food-safety and eco- relevance such as green food and organic food certificates [18–23]. However, their survey-based methods in eliciting these WTPs may have hypothetical bias, a severe disadvantage over revealed preference using actual sales data [24,25].

Because of the large transaction volume with numerous varieties and relatively stable 88-day shelf lives, apple is a good example to study the fresh food online market in China. China is the world’s largest apple producer, with 41.39 million tons in 2017 [26]. There are all kinds of varieties of apples, such as Fuji, Gala, Red delicious, Golden delicious, etc., as well as established places of origins and geographical indication, such as Tianshui in Gansu province, and Aksu in Xinjiang province [26]. In addition to domestic production, China also imported about 68,000 tons of apples in 2017, mainly from the United States, New Zealand and Chile [26]. Both intrinsic and extrinsic attributes for apples have been extensively studied by food scientists, such as Endrizzi et al. [27], Gatti et al. [28], and economists such as Manalo [29], Baker [30], Fotopoulos and Krystallis [31], Kim et al. [32], and Ceschi et al. [33]. The pricing strategy for Chinese online market is also studied [34]. These studies give us good guidelines in the valuation of apple attributes, although none of them addresses the online market issues.

The objective of this paper is to study factors affecting consumer online fresh food preferences reflected through the price. More specifically, we investigate factors affecting the price of fresh produce in online markets incorporating both product-related attributes and store-related attributes in a hedonic pricing model, using apple as an example and using market revealed preferences instead of stated preferences.

## Materials and method

### Attribute choice

Vast literature exists for studying the attributes of fresh fruits, especially apples. Attributes of fresh fruits can be divided into search attributes, experience attributes and credence attributes. Search attributes refer to attributes which can be known before purchasing, including price, size and package, related studies are often conducted by economists in a survey setting mimicking market purchasing situations [29–33]. Experience attributes refer to those who can only be known after purchasing or tasting, for apples, many lab-based consumer test results show that sweetness, juiciness, and crispiness significantly influence consumer’s preferences [27,28]. Credence attributes refer to attributes that can’t be known even after consumption, a typical example is organic labels, numerous literatures have shown importance of credence attributes in consumption behavior, Wang et al. [35], Massey et al. [36], Liu et al. [37], Rang and Paul [38] have reviewed motivations behind.

In this study, we use actual data observed from the online market that no quantifiable measurements about intrinsic attributes such as sweetness or crispiness are available. However, particular apple variety has unique intrinsic attributes, and we use this observable variable to represent them. Water core is another indicating a special physiological condition of apples produced in a certain environment resulting in sweeter taste than the same variety without this condition. As in most studies we also have the place of origin, with several apple producing provinces in China and importing source countries. Fruit size is also considered. Another important variable has the government certified green food or organic label. This label represents both food safety and ecofriendly, because overuse of pesticide and other chemicals is serious food safety concern in China, is shown make significant difference for consumption choice [39].

Additional store factors are also considered. Unique to online stores, previous consumer reviews provide good references to current consumers. Those scores represent reputations which is known to have significant influence on consumer’s willingness to pay [40,41]. In our data, we can observe scores for the specific product quality, for individual stores, for the service, and the logistics. Further, the web publishes the total count of written reviews that are separated into good reviews and bad reviews.

### Regression model

This study uses a hedonic pricing model to estimate the implicit price of each attribute of apples. A hedonic pricing model is a great tool to study the pricing of products with various attributes. A hedonic analysis is based on the hypothesis that consumers derive utilities from the qualities and characteristics of the goods they consume, and therefore the market price is the function of the implicit price of each attribute of the goods and thus reflects consumers’ preference [42]. Numerous studies have used the hedonic pricing model to study the pricing of food attributes [43–45]. However, the vast majority of them are about goods in traditional offline markets [46,47]. Only a few studies focus on the online food markets [48,49].

In our case, where apples are sold in online markets, the hedonic price function is the following:

$$Price=x\beta +\varepsilon =\beta_{0}+\sum_{k=1}^{15}\beta_{1k}{Origin}_{k}+\beta_{2}Watercore+\sum_{k=1}^{7}\beta_{3k}{Variety}_{k}+{\beta_{4}Greenororganic+\beta_{5}*Size+\beta_{6}*Productscore+\beta_{7}*Goodreview+\beta_{8}*Badreview+\beta_{9}*Video+\beta }_{10}*Storescore+\beta_{11}*Servicescore+\beta_{12}*Logisticscore+\sum_{k=1}^{2}\beta_{13k}*{Biweekly}_{k}+\sum_{k=1}^{7}\beta_{14k}{Variety}_{k}*Video+\varepsilon$$ (1)

Where *Price* is the unit price of each apple product (yuan/kg); *Origin*_*k*_ is a series of binary variables for different places of origin using Xinjiang province as the base; *Watercore* is a binary variable taking value 1 when the apple is water corn and 0 otherwise; *Variety*_*k*_ is a series of binary variables indicating varieties of apples using Fuji as the base; *Greenororganic* is a binary variable indicating whether the product has the special government certified green or organic label; *Size* is a continuous variable for the average size of the apple measured by the fruit diameter in millimeters (many stores don’t provide this measurement); *Goodreview* and *Badreview* are continuous variables representing the percentages of customer reviews for this product that are good and bad, respectively; *Storescore*, *Productscore*, *Servicescore* and *Logisticscore* are continuous variables representing the average of historical scores given by former buyers about the store, the product, the service, and the logistics, respectively; *Video* is a binary variable indicating whether the product has a video description online; and *Biweekly*_k_ is a series of biweekly dummies using the first two week period of our multi-period data as the base.

Some variables may have interactive effects on each other. For example, the *Goodreview* and *Badreview* may interact with the *productscore* because they both are about products. Also, the *video* may have different impact on different varieties, because each variety has its unique visual attributes in shape and color and video can highlight those and generate different effects to consumers. On the other hand, there is no economic and intuitive reasons to justify the interactive effects between the place of origin and those variables from customer reviews. For example, for the same size an variety, there is no reason for us to expect the *goodreview* effect is different for apples from Shandong versus from Hebei. Therefore, we have added the *video* and variety interactions as well as the reviews and product score interaction, but the latter was dropped because they are consistently insignificant for all model settings.

It is hypothesized that varieties and places of origins will have significant effect on the price; apples with water core feature and green or organic certificate have a price premium; observations with higher store score, product score, service score and logistic score will have higher prices; apple products with video description will have higher prices; good review will have positive effect on the price while bad review will have the opposite effect.

Promotion and discount are very common in the online market. It is often a strategy used by sellers to raise the price of the product and give a discount in order to attract customers without hurting the profit margin. If so, the promotion will have an effect on the price. However, promotion and listed price may be determined simultaneously, or joint endogenous. As a result, promotion cannot be used as an explanatory variable in the regression, otherwise all the regression results will be biased. In order to estimate the effect of the promotion on the price, a propensity score matching method will be used.

Due to missing value issues in empirical studies, especially using market observed data instead of surveyed data as in this study, there will be a tradeoff between the included number of explanatory variables and the size of observations. Hoping to see the large sample results and include more explanatory variables, we fitted three models to the regression equation. Model 1 includes the greatest number of explanatory variables but with the smallest dataset, whereas Model 3 uses the largest dataset while drops several variables that have too many missing values. Model 2 is in between. We will explain this in more details in the result section when we interpret the results from the three regressions.

### Propensity score matching method

The propensity score matching is a statistic technique developed by Rosenbaum and Rubin [50] to estimate a treatment effect by accounting for the covariates that predict receiving the treatment. This technique can reduce the bias due to confounding variables by comparing outcomes of observations that received the treatment and the ones that did not. Studies have used the propensity matching method to analyze market prices of essentials and food [51–53]. Because in observational studies the assignment of the treatment is not random, the estimation of the treatment is subject to selection bias. Matching is to use some criteria to mimic the randomization by creating comparable treatment and control groups on all observed covariates. Propensity score matching is to match by the probability of an observation belonging to the treatment group. In our model, $P_{i}=Prob(promotion=1|X_{i})=\exp\left(X_{i}\beta \right)/[1+\exp\left(X_{i}\beta \right)]$, where *X*_*i*_ are the explanatory variables in the hedonic pricing function above. The propensity score is the log odds: log[*Pi*/(*Pi*+1)]. After matching the propensity score across treatment and control groups, we can estimate the average treatment effect of promotion on the prices:

ATE=AveragePrice(promotion=1)−AveragePrice(promotion=0). (2)

### Data

Data of this study were collected via web crawler using Python from Jingdong (JD) E-commerce platform. We chose JD as the source of data because JD is the second largest e-commerce platform in China, and it has a good user interface that is relatively easy to extract information. The raw dataset had store pages observations across 6 weeks from 11/26/2018 to 12/31/2018. We have dropped those webpages that are not fresh apples for regular consumption, do not have a unique variety or place of origin, and those with missing important variable values. The final sample size is 8,200.

Table 1 shows descriptive statistics of selected variables. For price attributes, the average unit price is 34.58 yuan per kilogram, and about 25% of the observations have promotions; the average of the discount rate provided is about 3%. For product-related attributes, about 33% of the observations have the water core feature; and only 2.3% have a certified green or organic label. The average of the product score is 9.56 out of 10. Among locational variables, Shandong province, Shaanxi province, Gansu province, Xinjiang province and Sichuan province provide the most apple products, with 20%, 19%, 10%, 9%, and 9% shares, respectively, while imported apples from Chile, United States, Japan, New Zealand and France have small but visible shares of 1%, 1.5%, 0.45%, 2.8% and 0.18%, respectively. Among varieties, the vast majority, 60% of the observations are Fuji apples; and the second-largest apple variety is Red Delicious, with about 8.8% share. For store-related attributes, store score, service score, and logistic score are on average 9.68, 9.61, and 9.61, respectively. 44% of observations provided video description for the product. The average percentage of good reviews is 97%, and the average of bad reviews accounts for about 3%.

**Table 1:** Descriptive statistics of selected variables.

Variable	Description	Mean	Std
**Price attributes**			
**Price**	Unit price (yuan/kg)	34.58	52.24
**Promo**	Promotion = 1, no promotion = 0	25%	43%
**Maxdiscount**	Maximum discount rate if promotion = 1	31%	7.1%
**Product attributes**			
**Size**	Average size in mm	78.19	6.72
**Productscore**	Historical average score for the product	9.56	0.23
**Watercore**	Watercore = 1, not watercore = 0	0.33	0.47
**Greenororganic**	With green or organic certificate = 1, without certificate = 0	2.3%	15%
**Origin**	Series of binary variables for place of origin		
**Xinjiang**	= 1 if from Xinjiang province, otherwise = 0	9.3%	29%
**Sichuan**	= 1 if from Sichuan province, otherwise = 0	9.1%	29%
**Shandong**	= 1 if from Shandong province, otherwise = 0	20%	40%
**Shaanxi**	= 1 if from Shaanxi province, otherwise = 0	19%	39%
**Gansu**	= 1 if from Gansu province, otherwise = 0	10%	31%
**Yunnan**	= 1 if from Yunnan province, otherwise = 0	6.6%	25%
**Liaoning**	= 1 if from Liaoning province, otherwise = 0	1.3%	11%
**Henan**	= 1 if from Henan province, otherwise = 0	0.32%	5.6%
**Hebei**	= 1 if from Hebei province, otherwise = 0	0.13%	3.7%
**Shanxi**	= 1 if from Shanxi province, otherwise = 0	3.0%	17%
**Chile**	= 1 if from Chile, otherwise = 0	1.0%	10%
**U.S.**	= 1 if from U.S., otherwise = 0	1.5%	12%
**Japan**	= 1 if from Japan, otherwise = 0	0.45%	6.7%
**NewZealand**	= 1 if from NewZealand, otherwise = 0	2.8%	17%
**France**	= 1 if from France, otherwise = 0	0.18%	4.3%
**Other areas**	= 1 if from Other areas, otherwise = 0	15%	36%
**Variety**	Series of binary variables for variety		
**Fuji**	= 1 if Fuji apples, otherwise = 0	60%	49%
**Reddelicious**	= 1 if Red Delicious apples, otherwise = 0	8.8%	28%
**Goldendelicious**	= 1 if Golden Delicious apples, otherwise = 0	5.1%	22%
**Gala**	= 1 if Gala apples, otherwise = 0	2.0%	14%
**Greenapple**	= 1 Green apples, otherwise = 0	2.1%	14%
**Jonagold**	= 1 Jonagold apples, otherwise = 0	0.16%	4.0%
**RallsJanet**	= 1 Ralls Janet apples, otherwise = 0	2.3%	15%
**Other varieties**	= 1 other apple varieties, otherwise = 0	20%	40%
**Store attributes**			
**Storescore**	Historical average score for the store	9.68	0.12
**Servicescore**	Historical average score for service of the store	9.61	0.15
**Logisticscore**	Historical average score for the logistic speed	9.61	0.16
**Video**	= 1 if store web has video, otherwise = 0	44%	50%
**Goodreview**	Percentage of good reviews	97%	4.3%
**Badreview**	Percentage of bad reviews	2.8%	7.7%

## Results

The estimation is executed in STATA. Data in this study are obtained from online sales on JD platform with 8,200 total observations used in Model 3. However, each vendor on the platform may report different information on their store website, which results in the missing value of many variables. For example, are about half of the observations have missing values for the variables of store reviews. So, in Model 2 when we include the *goodreview* and *badreview* variables, we can only use 4,178 observations. Similarly, another half of these transactions that have store reviews don’t report fruit size, and we can only use the remaining 2.090 observations in Model 1 when we include all fruit size and store review variables.

The Breusch–Pagan test for heteroscedasticity is used which reported a p-value less than 0.0001, thus we rejected the null hypothesis of homoscedasticity. Therefore, we used heteroscedasticity-robust standard errors in our estimation instead.

The regression results are shown in Table 2. In the following, we will focus on results with at least 5% significance level. First, consumers have a clear preference on the place of origin. They prefer apples from some regions over others, because of the long-established reputation of quality from particular regions that are not measurable by any specific attributes. Among places of origins, in model 1, compared to Xinjiang province, on average, the price of apples from Sichuan and Yunnan provinces are 9.39 and 3.41 yuan lower, respectively, and the price of apples from Shaanxi province is 11.46 yuan higher. For imported apples, those from the United States, New Zealand are both significantly more expensive with 26.39 yuan and 92.28 yuan price premiums, respectively. In model 2, apples from Sichuan, Yunnan, and Shanxi provinces are 12.09 yuan, 7.99 yuan, and 5.89 yuan cheaper, respectively. Apples from Shaanxi and Hebei are 8.96 and 7.74 yuan more expensive, respectively. The prices of imported apples are all becoming significantly more expensive, with 178.02 yuan, 67.88 yuan, 59.71 yuan, 52.09 yuan and 49.67 yuan higher for New Zealand, Japan, Chile, France, and United States, respectively. The results in model 3 are similar to model 2: Hebei, Sichuan, Yunnan, Shanxi, and Gansu province have lower prices with 17.88 yuan, 14.22 yuan, 8.94 yuan, 6.60 yuan, and 4.66 yuan, respectively. Apples from Shaanxi province are still more expensive with 5.97 yuan price premium, and all imported countries have higher prices as in model 2. Overall, all imported apples are strongly preferred and can be sold with a large price premium over domestic apples. This is because apples have been grown widely in China as a traditional fruit, and only apples with superior quality embedded in newer varieties are imported, which can gain the price premium to justify the transportation and other transaction costs. New Zealand apples are the most popular ones in Chinese online market, not only for they receive the largest number of online purchases, 2.8% share from Table 1, but also the highest price premium among all imported apples. Among domestic apples, Shaanxi ranks on top, Xinjiang ranks next similar to Shandong and Liaoning, while Sichuan ranks at the bottom after Yunnan and Shanxi. These are consistent to the traditionally established regional reputation. Shaanxi has the best agronomic condition for apples and it is also the first region that Fuji apples were introduced to replace the indigenous varieties. Xinjiang and Shandong have similar climate suitable for apple production. However, Sichuan and Yunnan are warmer and wetter regions and their apple production are mostly on mountainous regions without homogenous quality.

**Table 2:** Regression coefficients of hedonic price analysis.

	Model 1 (With all variables, sample size 2,090)		Model 2 (Without Size, Sample size 4,178)	Model 3 (Without Size, Good-review & Badreview, Sample size 8,200)	
Sichuan		-10.55*** (2.04)	-12.09*** (1.21)		-13.94*** (1.27)
Shandong		0.44 (3.15)	-.66 (2.64)		-1.91 (1.90)
Shaanxi		11.58*** (3.38)	8.96** (3.54)		6.60** (2.64)
Gansu		5.23 (3.54)	-.77 (2.40)		-4.74** (2.27)
Yunnan		-2.61* (1.73)	-7.99*** (1.45)		-8.89*** (1.79)
Liaoning		NA	-.52 (3.71)		-2.79 (2.91)
Henan		3.43 (5.17)	1.69 (3.65)		-2.91 (2.39)
Hebei		NA	7.74* (3.98)		-7.70 (5.49)
Shanxi		-0.95 (3.59)	-5.89*** (2.25)		-6.38*** (1.62)
Chile		NA	59.71*** (9.52)		48.62*** (7.09)
US		26.90*** (8.09)	49.67*** (7.32)		40.31*** (5.18)
Japan		NA	67.88*** (9.14)		93.63*** (16.13)
New Zealand		90.71*** (29.56)	178.02*** (13.65)		150.17*** (9.22)
France		NA	52.09*** (11.78)		46.86*** (8.45)
Other areas		13.36** (6.64)	6.15* (3.29)		8.11*** (2.08)
Watercore		5.51* (3.18)	4.73* (2.76)		2.74 (1.98)
Red delicious		-3.91** (1.69)	-5.25 (1.76)		3.57** (3.74)
Golden delicious		-8.59*** (2.73)	1.19 (1.18)		1.32 (1.23)
Gala		-3.55 (5.45)	-45.17*** (10.65)		-32.85*** (6.69)
Green apple		-8.96*** (3.17)	-12.71*** (2.55)		-2.47 (3.59)
Jonagold		-10.51*** (5.08)	-4.76 (4.86)		-5.24 (3.88)
Ralls Janet		18.70*** (1.97)	-6.03 (2.07)		-5.75*** (1.60)
Other variety		4.76*** (1.90)	8.53*** (1.99)		16.19*** (1.85)
Green or organic		8.80*** (2.71)	7.26*** (2.02)		8.34*** (1.57)
Size		0.38*** (0.08)			
Productscore		-6.96** (2.99)	4.24 (4.98)		-3.90 (2.75)
Good review		-0.12 (13.43)	0.35** (0.14)		
Bad review		0.17* (0.098)	0.12 (0.073)		
Video		1.94* (1.09)	0.47 (1.44)		0.57 (1.14)
Video*Red Delicious			14.79*** (4.03)		6.40* (3.43)
Video*Golden Delicious		11.45*** (4.17)			
Video*Greenapple		19.09*** (3.64)			55.77*** (12.30)
Video*RallsJanet			9.90*** (2.76)		9.78*** (2.97)
Video*Other		13.19** (5.29)			
Storescore		32.72*** (7.53)	29.05*** (10.54)		43.05*** (8.63)
Servicescore		-0.10 (6.83)	-8.12 (10.20)		-38.78*** (8.97)
Logisticscore		-4.86 (5.43)	-3.57 (8.20)		16.62*** (7.25)
Biweekly 2		3.62** (1.51)	2.84** (1.46)		0.75 (1.24)
Biweekly 3		4.32** (1.81)	5.68*** (1.75)		4.04*** (1.22)
Constant		-218.02*** (47.32)	-226.51*** (46.29)		-143.77*** (35.59)
R-squared		0.0848	0.3597		0.3046
F		47.66***	34.06***		31.10***
Observations		2,090	4,178		8,200

Note: Heteroscedasticity robust standard errors are reported in the parenthesis.

*, ** and *** denote the significant levels at 10%, 5% and 1%, respectively. NA’s are used for origin dummies of Liaoning, Hebei, Chile, Japan and France, as they have no apple transactions in the smaller sample used in model 1.

Some measurable qualities are also found important for consumers, and their preferences are reflected on prices. In models 1 and 2, on average, apples that are watercore are 5.96 and 4.6 yuan more expensive than apples without watercore feature, showing it is a desirable attribute. It becomes insignificant in model 3, which can be a result of missing variable caused biases. There is also a green or organic certificate price premium of 7 to 9 yuan across the three model specifications. This food safety and ecofriendly labels are an important positive feature that consumers prefer, which is consistent with [10]. Larger apples are also preferred as expected. On average, the price of apple increases by 0.37 yuan if the size of the apple increases by 1 millimeter in diameter, or 3.7 yuan per centimeter.

Apples of different varieties are quite different in taste and texture and also appeal to consumers differently. Compared to Fuji apple as the base, other variety and Ralls Janet have a consistent price advantage. This is because small varieties pooled into the category of others tend to be new and high quality varieties, so is Ralls Janet. This finding is supported by the arguments that consumers’ tastes are evolving overtime and that newer varieties emerged in market catch their updated preferences and so WTP [54,55]. Golden delicious has a similar price as Fuji. Gala price tends to be lower, with significant measures in models 2 and 3. Jonagold also tends to be cheaper than Fuji. Red Delicious and Green apple show inconsistent signs in different models. This change in signs is because the number of observations for these varieties depends on the variables that we exclude in models 2 and 3 due to missing observations. If we trust model 1, these two varieties are less preferred than Fuji, which is consistent with observations in the world because they are the oldest varieties [55].

Other than their own intrinsic attributes, apples sold online have special attributes that are not available for apples sold offline. These attributes also provide particular value to consumers and causing them to pay higher price premiums to apples with these attributes. Observations with video description are 4.42 yuan higher than ones without video description in model 1, suggesting that having a video description of the product, Fuji variety by default, has positive effect on the price. Unlike busy shoppers in grocery stores, online shoppers have more time to watch such videos as they do online shopping and see videos as a value-added service. Although we see significant positive effects of video interaction with different varieties in different model specifications, its interaction with Gala, and Jonagold is consistently insignificant in all models. This means video works well for apples with unique visual characteristics, such as green color for Green apple, yellow color for Golden delicious, dark red color for Red delicious, but not for general looking apples.

Reviews left by previous customers are valuable to future customers [56,57], which is a unique feature for online stores. Studies [57–59] have shown that customer reviews about the product and deliver service can influence shoppers’ shopping choices, and are reflected to the price. In our study, store score shows a significant positive effect on the price with 30.15, 27.31 and 37.91 yuan higher for each additional store score in models 1,2 and 3, respectively. These are rather high marginal values for apples, and they provide strong incentives to online store to improve their services to earn higher store scores given by customers. The ratio of good reviews shows a positive impact only in model 2. Logistic score also shows a positive effect only in model 3.

The higher biweekly dummies for the latter two periods are because it gets closer to the Christmas and New Year season and shows an overall seasonality, not related to product or store attributes.

**Fig 1:**
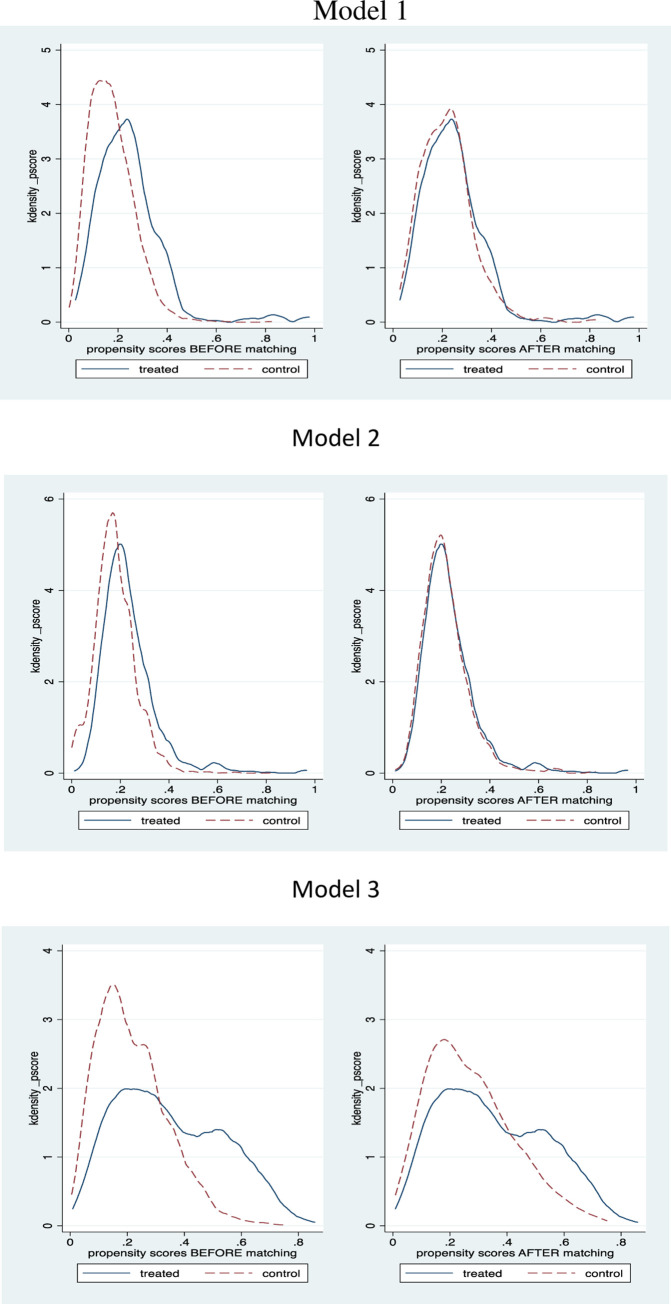
Propensity score before and after matching.

We also estimated the average treatment effect (ATE) of promotion on the prices using the propensity score matching method (Table 3). The density distributions of prices are plotted in Fig 1 for alternative models. They consistently show that the matched dataset has treated groups and control groups are much more similar than the unmatched data. In model 1, the price of apple products with promotion is about 4.41 yuan cheaper than ones without promotion. However, the effect of the promotion is not significant in model 2 and model 3 when large datasets are used. This means the apples that are sold under discount promotions tend to have similar listed prices with those that are not under discount promotions with our large sized observations. For the subset of observations, the former shows lower listed prices that the latter, or we can say those lower prices apples correlated with running price discount promotions. Combining the two, this does not support the suspicion that stores first raise the price to a high level and run a discount creating a false impression of low price to attract consumers. Instead, the higher priced apples do not tend to run such discount promotions while the lowered priced apples do, showing a fiercer price competition among the low-end apples.

**Table 3:** Average treatment effect of promotion on the prices.

Model	Number of Observations	Mean Difference	P-value
Model 1	2,090	-4.41	0.000
Model 2	4,178	-.85	0.652
Model 3	8,200	.95	0.538

## Discussion and conclusion

Our study uses hedonic pricing models to analyze the implicit prices of apple attributes in online markets. Unlike many food attribute valuation studies using survey-based stated preference data, we use actual online sale data with detailed attributes to show the revealed preference that effectively avoids hypothetical bias. As price is the indicator of the market supply and demand equilibrium, for a widely available product in market with some attribute differences, a higher price means the corresponding attribute is preferred by consumers and is in high demand not perfectly substitutable by the same product without that attribute.

Using fresh apple sold on JD platform in China as an example, we found that consumers show preferences on both intrinsic food attributes and online market attributes through the fact that both product-related and store-related attributes affect price in online markets. Such findings will provide the food industry useful information to deal with the new and fast-growing online market channels.

Varieties and places of origins are both important factors affecting the price. Our Ralls Janet, Fuji, and other smaller varieties have the highest price, which is supported by literature [53,54] because newer varieties emerged in market catch consumer’s updated preferences. There are also significant price premiums for imported apples and for apples that are produced from certain domestic regions with established reputations. In particular, apples from New Zealand are the most popular apples among all imported ones and enjoy the highest price premiums. Two reasons can explain these. Firstly, because apples have been grown widely in China as a traditional fruit, consumers are quite discerning about apples, and thus only apples with superior quality grown under ideal conditions can receive consumers’ recognition and gain the price premium. Foreign exporting countries with different climate and Shaanxi and Xinjiang provinces having the best agronomic condition yield apples of higher quality. Secondly, these countries have advanced production technologies that ensure the homogenous the apple qualities, so do the two Chinese regions that are outside the traditional apple areas and established more recently with newer commercial technologies.

Organic or green certificates have a significant price premium, resulting from consumers’ concerns of food safety and the food-environment interaction. Chinese consumers are especially concerned about excess chemical use in food production, in particular pesticide residuals in apples [60], because there is in general lack of reinforcement on the safe level of chemical applications. Thus, they turn to food with official certifications of organic or green labels [61].

Price discount is a common promotion method used by sellers. Our results shows that this method is used more by lower priced apples than by higher prices apples. This shows a fiercer price competition among low-end products than high-end products. It does not support the suspicion that stores first raise the price and run a discount causing a false impression of low price to attract consumers.

Price discount promotion is not a tool adopted by higher-priced apple sellers. The results can shed lights to online market price scheme, for example, by comparing the cost of producing green or organic food product with the price premium they can bring. This finding provides long term incentives for small venders to sell good products and provide sound services.

Online video and reviews about the stores and products from previous customers are a different set of factors to affect the price. These factors are only available for online market, and are missing in existing fresh food market literature. The use of video descriptions for apples can generate price premiums for apples that have unique appearances. Especially, Red Delicious, Golden Delicious, Green Apples have unique color, and stores showing a video online can receive higher price than stores selling the same apples without a video. This new finding tells online vendors to make best use video and other new web visual tools to promote their relevant products. Our study also show that reviews left by previous customers are valuable to future customers, which is consistent to existing in non-food market studies [57–59]. They show that previous customer reviews about the product and deliver service can influence shoppers’ shopping choices. In our study, store and logistic scores show a significant positive effect on the price. This provides strong incentives to online store to improve their services to earn higher store scores given by customers.

There exist some limitations of this study. Although using actual market data can completely avoid hypothetical bias, we don’t have individual shopper’s characteristic information and thus cannot explore any heterogeneity in consumer preference nor its causal factors. The shorter data period also missed the seasonal cycle and storability attribute of different varieties of apples. These weaknesses call for future studies using data with longer period and combined survey with actual online sales.
